# Epigenetic Variability Among Saffron Crocus (*Crocus sativus* L.) Accessions Characterized by Different Phenotypes

**DOI:** 10.3389/fpls.2021.642631

**Published:** 2021-03-04

**Authors:** Matteo Busconi, Elisabeth Wischnitzki, Marcello Del Corvo, Licia Colli, Giovanna Soffritti, Lorenzo Stagnati, Silvia Fluch, Eva Maria Sehr, Marcelino de los Mozos Pascual, José Antonio Fernández

**Affiliations:** ^1^Faculty of Agriculture, Food and Environmental Sciences, Research Centre BioDNA, Università Cattolica del Sacro Cuore, Piacenza, Italy; ^2^Centre for Health and Bioresources, AIT Austrian Institute of Technology, Tulln, Austria; ^3^Centro de Investigación Agroforestal de Albaladejito, Instituto Regional de Investigación y Desarrollo Agroalimentario y Forestal, Cuenca, Spain; ^4^IDR-Biotechnology and Natural Resources, Universidad de Castilla—La Mancha, Albacete, Spain

**Keywords:** *Crocus sativus*, saffron, epigenetics, epigenomics, DNA methylation, methylation-sensitive restriction enzyme-sequencing, differentially methylated regions, flowering

## Abstract

This work represents the first epigenomic study carried out on saffron crocus. Five accessions of saffron, showing differences in tepal pigmentation, yield of saffron and flowering time, were analyzed at the epigenetic level by applying a methylation-sensitive restriction enzyme-sequencing (MRE-seq) approach. Five accession-specific hypomethylomes plus a reference hypomethylome, generated by combining the sequence data from the single accessions, were obtained. Assembled sequences were annotated against existing online databases. In the absence of the *Crocus* genome, the rice genome was mainly used as the reference as it is the best annotated genome among monocot plants. Comparison of the hypomethylomes revealed many differentially methylated regions, confirming the high epigenetic variability present among saffron accessions, including sequences encoding for proteins that could be good candidates to explain the accessions’ alternative phenotypes. In particular, transcription factors involved in flowering process (MADS-box and TFL) and for the production of pigments (MYB) were detected. Finally, by comparing the generated sequences of the different accessions, a high number of SNPs, likely having arisen as a consequence of the prolonged vegetative propagation, were detected, demonstrating surprisingly high genetic variability. Gene ontology (GO) was performed to map and visualize sequence polymorphisms located within the GOs and to compare their distributions among different accessions. As well as suggesting the possible existence of alternative phenotypes with a genetic basis, a clear difference in polymorphic GO is present among accessions based on their geographic origin, supporting a possible signature of selection in the Indian accession with respect to the Spanish ones.

## Introduction

Saffron crocus (*Crocus sativus* L.) is a sterile triploid (2*n* = 3× = 24) species whose botanical origin is still not clear. Initially assumed to be of autotriploid origin, subsequently other evidences supported alloploidy as the most probable mechanism of origin, with *Crocus cartwrightianus* Herb. being one of the two most likely ancestors (Fernández, 2004). Finally, and contrary to the allopolyploid hypothesis, the most recent findings support the auto-triploid origin starting from *C. cartwrightianus* (Nemati et al., 2018, 2019). Since the first domestication that presumably occurred more than 3,500 years ago, the species has been vegetatively propagated via the yearly production of new corms (Fernández, 2004). Corm multiplication does not generate genome variations, apart from some spontaneous mutations that are not easy to detect in a triploid saffron population. Consequently, the presence of genetic variability among accessions with similar or different geographic origin has been debated for many years (Rubio Moraga et al., 2009; Siracusa et al., 2013). In contrast, phenotypic variation, such as differences in the number of stigmas or appearance of tepals, is frequently observed in the field by researchers and saffron producers. Interestingly, observed phenotypic variations are occasionally unstable and can change between growing seasons (Fernández, 2004). In a preliminary characterization of 50 saffron accessions of the WSCC (World Saffron and Crocus Collection), characters related to phenology, floral morphology and saffron production were measured and high levels of variation were detected (De Los Mozos Pascual et al., 2010). These observations raise the question about the origin of such phenotypic variability. If not due to genomic variation (given the low mutation rate in corm multiplication), epigenetics could be a possible source of these alternative phenotypes, given that gene expression can be influenced by both genetic and epigenetic changes.

As recently reviewed (Springer and Schmitz, 2017), several studies have provided evidence on the role of epigenetics in creating inheritable variation and in contributing to important traits in many plant species. Epigenetic marks are defined as a series of chemical modifications of DNA and histones, but in plants, most studies have mainly focused on DNA methylation, which presents inheritance mechanisms and is relatively simple to investigate genome-wide (Seymour and Becker, 2017). The most frequent mark of DNA methylation is 5-methylcytosine (5-mC), which in plants can be methylated at different sites containing CG, CHG and CHH (H represents any base but G) sequences (Wischnitzki et al., 2015). The extent of cytosine methylation varies among plant species and along the chromosomes, but the functional consequences for gene expression are strongly influenced by the location of 5-mCs relative to the gene. In plants, methylation marks can also vary according to the cell, tissue, and organ in the vegetative and reproductive phases of a plant’s life cycle (Widman et al., 2014). Notably, methylation patterns acquired to cope with stresses are often transmitted to subsequent generations (Zhang et al., 2013). Most of the current knowledge concerning plant DNA methylation has been obtained from studies carried out on *Arabidopsis thaliana*, but investigations on different and agronomically important species have also been published (Wang et al., 2014; Seymour and Becker, 2017; Springer and Schmitz, 2017), often associating aberrant phenotypes with specific epigenetically regulated loci (Ong-Abdullah et al., 2015).

There is currently limited knowledge concerning saffron, and genetic and epigenetic studies are far behind those carried out on other crops. Several years ago, in a survey of more than 100 accessions of the WSCC collection, a very low level of genetic variability was detected using 12 AFLP primer combinations, but surprisingly high epigenetic variability was revealed using just three MS-AFLP primer combinations (Busconi et al., 2015). Interestingly, despite at least 3 years of co-cultivation and vegetative propagation in the same field, accessions with different geographic origins still possessed different epigenotypes, making it possible to separate the accessions of different origin, at least in some cases. In another study, based on differences in the MS-AFLP profile of different parts of the flower, the possible utility of epigenetics for detecting stamen adulteration of saffron production was suggested (Soffritti et al., 2016). Finally, Busconi et al (2018) evidenced that epigenetic structure in saffron is highly stable and can stably persist over the years, supporting again the possible involvement of epigenetics in determining alternative saffron phenotypes.

Given that the gene body in plants is characterized by rather low methylation levels (hypomethylation) and that, in contrast, cytosine methylation is most frequent in repetitive elements (e.g., transposable elements), studies of the hypomethylome can provide interesting information about the genes and regulatory regions subjected to epigenetic regulation and variation. Several methods have been developed to study the plant hypomethylome, one of which is the methylation-sensitive restriction enzyme-sequencing (MRE-seq) approach, MRE-seq (Wischnitzki et al., 2015). This method has been applied successfully to different plant species including saffron, making it possible, even in the absence of a reference genome and low coverage of the generated sequences, to isolate and sequence genes and regulatory elements, representing 26 and 47% of the total reads, respectively (Wischnitzki et al., 2015). To gain a deeper insight into the epigenetics of saffron, the current study compared the epigenetic profile of five phenotypically different, but genetically similar by AFLP analysis (Busconi et al., 2015), accessions from the WSCC germplasm using MRE-seq focusing on differentially methylated regions.

## Materials and Methods

### Plant Material

Five accessions (Table 1) from the WSCC (World Saffron and Crocus Collection, located in the *Centro de Investigación Agroforestal de Albadalejito*, Cuenca, Spain) germplasm showing stable alternative phenotypes were selected for the study. The five accessions were received at WSCC between 2006 and 2008, and they were co-cultivated in the same field for at least 6 consecutive years before the analysis. The experimental farm of Cuenca is located at an altitude of 950–1,000 masl. The edaphic characteristics of the fields are those common for the area, with sandy loams, alkaline pH (7.6–8.4), normal electrical conductivity (<400 mmhos/cm) and a low organic matter content (1–2.5%). The phenotypic evaluation of the accessions is reported (Supplementary Table 1).

**TABLE 1:** Accessions considered for the study.

Accession	Geographic origin	First sowing in the WSCC^*a*^	Phenotype
BCU001610	Cuenca (Spain)	2006	High saffron production
BCU002519	Albacete (Spain)	2008	Low saffron production
BCU001637	Albacete (Spain)	2006	Early flowering time
BCU001668	Teruel (Spain)	2006	Late flowering time
BCU001754	Srinagar (India)	2007	Different pigmentation of tepals

*For each sample the geographic origin (province and country), the year of the first cultivation in the fields of the germplasm collection in Cuenca and the alternative stable phenotypes are reported. ^a^World Saffron and Crocus Collection.*

The chosen accessions were planted using a randomized block design with three replicates per accession. All the accessions had previously been analyzed at the genetic level, revealing extremely low genetic variability (Busconi et al., 2015). The epigenetic analysis was carried out on DNA extracted from leaves collected at the end of the vegetative period, at the beginning of May 2015. For each accession, leaves of ten independent plants were pooled to give a global view of the accession and not of a single plant. After sampling, leaves were lyophilized and preserved at −80°C until analysis. DNA extractions were carried out using the commercial kit GeneElute Plant Genomic DNA Miniprep Kit (Sigma-Aldrich) according to the manufacturer’s instructions with one minor modification. In detail, we added 4% w/w of PVP (polyvinylpyrrolidone; Soffritti et al., 2016) to each sample during DNA extraction to facilitate the removal of secondary metabolites, such as polyphenols, and to produce a purer DNA for the subsequent analysis.

### Methylation-Sensitive Restriction Enzyme Sequencing (MRE-seq)

To carry out the methyl filtration enrichment of gene-related sequences, genomic DNA was digested using the restriction enzyme *Hpa*II. This enzyme was selected because it is able to achieve very good gene space coverage and very good depletion of transposable elements, as also reported in Wischnitzki et al. (2015). In brief, the steps of the MRE-seq analysis are: (1) digestion of 300 ng of genomic DNA with the restriction enzyme *Hpa*II and contemporary ligation of double strand adaptors to the generated fragments; (2) PCR amplification of the adaptor-ligated DNA; (3) removal of the adaptor sequences by *Pme*I digestion (the sequence of the adaptor includes the rare cutting site of the enzyme GTTTAAAC). This step is important to increase the length of the informative sequences; (4) sequencing of the generated DNA fragments was done on an Illumina MiSeq platform (300 bp paired-end reads). All steps were carried out exactly as reported in detail in Wischnitzki et al. (2016).

### Quality Control and Processing of Sequencing Data

Following the protocol and guidelines for MRE-seq data analysis (Wischnitzki et al., 2016), all sequence reads were cleaned to guarantee high quality (HQ) data by removing adaptor reads, ambiguous reads, low quality regions (Q30), and short sequences (<50 bp) using bowtie2 (version 2.0.5; Langmead and Salzberg, 2012), samtools (version 1.2; Li et al., 2009), and bedtools (version v2.24.0; Quinlan and Hall, 2010). Then, sequence reads were analyzed using the same pipeline for their origin from potential repetitive elements [RE-dat database (Nussbaumer et al., 2013)], ribosomal data (using ribosomal sequences from NCBI and unpublished in-house data), chloroplast and mitochondrial DNA. Because of the absence of a reference genome for saffron crocus, the rice genome *Oryza sativa* ssp. *japonica* release 7 (Kawahara et al., 2013) was used as a reference.

All the HQ read pairs were tested to detect whether their ends overlapped with a minimal overlap of 10 bp and a maximal overlap of 250 bp using Flash (version: 1.2.10; Magoc and Salzberg, 2011).

These tools were mostly executed through perl scripts with the parameters as suggested by Wischnitzki et al. (2016). The corresponding perl scripts are provided in the following github repo: https://github.com/evasehr/MREseq_AnalysisWorkflow.

### *De novo* Assembly

The *de novo* assembly for each accession and for the combined dataset was performed using Trinity (version: 2.1.1; Haas et al., 2013) with default parameters and a minimal contig length of 100 bp. The resulting contigs were evaluated by mapping the high-quality reads used for the assembly to the assembled contig sequences using bowtie2 with default settings (version 2.0.5; Langmead and Salzberg, 2012), and only contigs consisting of at least five reads were retained. The resulting files were further processed using samtools (version 1.2; Li et al., 2009) and bedtools (version v2.24.0; Quinlan and Hall, 2010). Corresponding perl scripts can be found in the above mentioned github repo.

### Functional Annotation and Classification

Functional annotation of the *de novo* assembled contigs was performed using blastx for sequence similarity searches against NCBI’s non-redundant sequence database (version 2.2.28+). Similarities were considered significant with *E*-values ≤ 1e-5. Protein domains and motifs were detected by applying InterproScan (Jones et al., 2014). Gene Ontology (GO) terms were assigned using Blast2GO (Conesa et al., 2005; Conesa and Götz, 2008). If applicable, KEGG pathways^1^ were assigned to the *de novo* assembled contigs.

All contigs that could not be annotated by similarity to NCBI’s nr database were subjected to an additional similarity search using blastn against NCBI’s nucleotide database (*E*-value ≤ 1e-5).

All sequences were further compared with available saffron sequences. FASTA sequences available at NCBI from different databases (EST, nucleotide, protein) for *Crocus sativus* were obtained together with the published transcriptome sequences (Jain et al., 2016). All assembled contigs were compared to those datasets using Blast (version 2.2.28) with an *E*-value threshold of 1e-5.

### SNP Identification

To identify single nucleotide polymorphisms (SNP), the per base coverage was estimated using mpileup included in the samtools software package (version 1.2; Li et al., 2009) with the parameter –AB. All SNPs and Indels were identified using picard tools (version 2.2.1;^2^) and HaplotypeCaller of GATK (version 3.5-0-g36282e4; McKenna et al., 2010; DePristo et al., 2011). The difference of each position was calculated based on the base occurrences at that position. The base counts for each position were regarded as vectors and normalized for each trait with the coverage of the position resulting in comparable vectors. The resulting difference score represented the sum of the absolute values over all base possibilities. It ranged from 0 to 200. True SNPs were considered in the presence of a difference score higher than 180.

### Population Structure Analysis

Analysis of population structure between the five different accessions was carried out using different statistical approaches. First a principal component analysis (PCA) was performed within the statistical environment R using the library *SNPrelate* (Zheng et al., 2012) by calculating the genetic covariance matrix from genotypes, computing the correlation coefficients between sample loadings and genotypes for each SNP, estimating SNP eigenvectors (loadings) and projecting samples onto the plot.

Population structure was then assessed by the maximum-likelihood based approach implemented in the Admixture software v1.2 (Alexander et al., 2009). This software uses genotype data to cluster individuals into subgroups, with the expected number of subgroups (K) specified beforehand. Analyses were run for *K*-values ranging from 2 to 5 under the assumptions of Hardy–Weinberg equilibrium, complete linkage equilibrium and under the “unsupervised” mode (i.e., no prior information on the population of origin of the individuals). To identify the best fitting number of hypothetical populations, for each *K*-value, we used both fivefold cross-validation error values and the number of iterations needed to reach convergence. Results were visualized using the R package *pophelper* (Francis, 2017).

## Results

### Quality Control, Processing and *de novo* Assembly

Original sequencing reads were pre-processed via the following steps: removal of PhiX contamination, removal of ambiguous bases on both read ends and reads including mainly ambiguous sites, removal of low quality base pairs (Q30), removal of reads shorter than 50 bp (HQ reads in Table 2). The overall contamination of the sequencing reads with phiX was extremely low (approximately 0%) as very few reads (759) were identified as the phiX sequence. The HQ reads were further checked to test whether they might be derived from the following DNA sources: repetitive and transposable elements, ribosomal sequences, chloroplasts and mitochondria (Table 2). The presence of reads from these sources in HQ reads was extremely low, usually accounting for less than 1%, with just a few exceptions. In particular, the percentage of reads recognized as belonging to repetitive elements was usually below 0.1%. At the end of this step, a very high percentage of the original reads, ranging from 97.7% for the forward sequencing run (R1) of BCU001610 to 92.4% for the reverse sequencing run (R2) of BCU001668, could be used for further analyses. The filtered HQ reads, R1 and R2 of each accession, were subsequently checked to search for overlaps (Table 3). For each accession, from 79.7 to 82.3% of reads overlapped consistently.

**TABLE 2:** Filtering results.

Sample ID	BCU001610		BCU001637		BCU001668		BCU001754		BCU002519	
Total	5,554,424		6,185,734		6,704,196		5,760,678		5,911,990	
R1/R2	R1	R2	R1	R2	R1	R2	R1	R2	R1	R2
Reads per direction	2,777,212	2,777,212	3,092,867	3,092,867	3,352,098	3,352,098	2,880,339	2,880,339	2,955,995	2,955,995
HQ reads	2,734,395	2,633,282	3,043,603	2,921,853	3,285,078	3,170,443	2,828,994	2,731,853	2,898,702	2,795,135
Repetitive	1,533	1,695	2,198	2,404	5,214	5,214	1,741	1,959	3,364	3,556
%	0.06%	0.06%	0.07%	0.08%	0.16%	0.16%	0.06%	0.07%	0.12%	0.13%
Ribosomal	2,572	3,510	5,001	6,974	11,413	11,244	4,744	6,506	7,984	10,724
%	0.09%	0.13%	0.16%	0.24%	0.35%	0.35%	0.17%	0.24%	0.28%	0.38%
Chloroplast^†^	4,394	4,186	1,484	1,435	37,574	37,296	3,884	3,874	1,102	1,088
%	0.16%	0.16%	0.05%	0.05%	1.14%	1.18%	0.14%	0.14%	0.04%	0.04%
Mitochondrion^†^	11,322	10,681	15,661	14,815	18,950	18,045	12,602	11,972	12,705	12,069
%	0.41%	0.41%	0.51%	0.51%	0.58%	0.57%	0.45%	0.44%	0.44%	0.43%
Filtered HQ reads	2,714,574	2,613,210	3,019,259	2,896,225	3,211,927	3,098,644	2,806,023	2,707,542	2,873,547	2,767,698
**%**	**97.7%**	**94.1%**	**97.6%**	**93.6%**	**95.8%**	**92.4%**	**97.4%**	**94.0%**	**97.2%**	**93.6%**

*An average of 0.1% of the high quality (HQ) reads were derived from repetitive regions, 0.24% from ribosomal sequences, 0.31% from the chloroplast genome and 0.47% from the mitochondrial genome. ^†^Due to the lack of a published genome sequence and to the high conservation between plastid genomes between species, the sequences of another monocot O. sativa were used as references for both mitochondrion and chloroplast.*

**TABLE 3:** Overlapping read paired ends.

Sample ID	HQ pairs	Overlapping	%
BCU001610	2,590,409	2,125,287	82.0%
BCU001637	2,870,947	2,289,202	79.7%
BCU001668	3,052,740	2,441,425	80.0%
BCU001754	2,679,665	2,205,532	82.3%
BCU002519	2,738,988	2,219,177	81.0%

*On average 81% of read pairs were considerably overlapping (minimum 10 bp) and could be merged. This number is comparable to other studies.*

Overlapping reads were used for a *de novo* assembly. The assemblies were performed not only separately for each single dataset and accession but also by combining all five datasets to create a reference hypomethylome representing all samples (Supplementary Figure 1 and Table 4). Sample-specific assemblies were subsequently used to identify whole contigs representing differentially methylated regions specific for single accessions. The reference hypomethylome comprised 43,897 contigs representing 86.2% of all reads. The assembled contigs of the reference assembly had an average length of 320 bp, ranging from 103 to 1443 (Supplementary Figure 1 and Table 4). Data for the single assemblies are reported in Table 4. The reference assembly was a good representation of the separate datasets for each accession, as more reads could be identified to match the reference than the individual assemblies. Those additional reads might belong to contigs with a low number of matching reads that might have been excluded as potential artifacts in the quality check after the initial assembly process due to very low coverage in the individual samples.

**TABLE 4:** Overview of the assembly statistics.

Dataset	Contigs	% Reads included	Average reads per contig	Average contig length
BCU001610	40,392	80.9%	107	313
BCU001637	33,694	79.6%	140	318
BCU001668	46,924	80.3%	108	318
BCU001754	34,016	83.3%	135	320
BCU002519	32,820	75.8%	130	318
Reference Hypomethylome	43,897	86.2%	564	320
Reads included in reference hypomethylome by accession	BCU001610	87.34%		
	BCU001637	86.98%		
	BCU001668	85.97%		
	BCU001754	89.34%		
	BCU002519	81.73%		
Average percentage	/	86.27%		

### Sequence Annotation

The assembled 43,897 contigs of the reference hypomethylome were compared to the NCBI redundant protein sequences NR and nucleotide NT databases. All searches focused on available *Viridiplantae* sequences. Additionally, the sequences were compared to the protein and genome sequences of rice *O. sativa* L. as the best annotated monocot species to date. In total, 11,569 contigs (26.35%) could be annotated as at least partially protein coding. This is consistent with previous studies in other organisms showing a preference of hypomethylated regions for coding regions (Wischnitzki et al., 2015). GO terms and KEGG pathway information were also inferred for the protein coding sequences. The general results of this annotation step are reported in Table 5. More detailed results are reported in Supplementary Table 2 in the active sheets NR, NT, *O. sativa* genome, GO and KEGG. In the NR and NT sheets, the accession number of the best hit and the corresponding description are reported for each contig showing a positive hit with existing plant sequences, as well as the identification of the contig itself, along with some statistical parameters such as percentage of identity and the *E*-value as provided by blast analysis. Comparison with the rice genome showed a high sequence similarity between both monocot species for the detected sequences. As there is currently no saffron genome available, this approach could give an additional indication of genes that are potentially close to the identified hypomethylated region. However, those associations need to be validated either experimentally or with a *C. sativus* genome sequence. Seven hundred and ninety-three (793) contigs showed similarity with the reference genome of *O. sativa japonica* and were mapped along the 12 different chromosomes of the rice genome. After removing the duplicated values, i.e., different contigs mapping on the same rice genome region, 504 contigs remained with an average of 42 contigs per chromosome, ranging between 23 and 69 contigs mapping on rice chromosome 8 and 1, respectively. For each contig the corresponding rice gene was provided. For the non-coding contigs that could be identified in the rice genome, the closest gene was inferred and reported (Supplementary Table 2).

**TABLE 5:** Contigs of the different assemblies presenting similarity with published sequences (upper part) and, more specifically, published saffron sequences (lower part).

	Total contigs	NCBI NR protein	NCBI NT nucleotide		*O. sativa* genome		*GO terms*	*KEGG pathways*
**Reference hypomethylome**	43,897	11,569	2,563		793		7,975	2,736
	**Total contigs**	**Saffron EST**		**Saffron nucleotide**		**Saffron protein**		**Saffron transcriptome**
BCU001610	40,392	272		62		233		13,170
BCU001637	33,694	295		70		269		15,070
BCU001668	46,924	335		85		291		15,811
BCU001754	34,016	352		84		312		16,820
BCU002519	32,820	206		55		173		10,146
Reference hypomethylome	43,897	366		88		322		17,523

*The number of contigs associated with GO terms and KEGG pathways is also presented.*

Our results showed that the number of GO terms associated with each single contig could be extremely variable e.g., contig TRINITY_DN10001_c0_g1_i1 was associated with a single GO term (GO: 0044464) and contig TRINITY_DN4528_c0_g4_i1 was associated with 35 different GO terms (Supplementary Table 2). Several contigs encoded for known proteins for which it was possible to identify a KEGG pathway and to provide a protein description in the pathway (Supplementary Table 2). Among the others, it was possible to detect pathways involved in the metabolism of carbohydrates, energy, lipids, nucleotides, amino acids, glycans, cofactors and vitamins, terpenoids, and polyketides and other secondary metabolites. Supplementary Table 3 lists the different contigs encoding for the same enzyme for each KEGG pathway. The most represented pathways were those of carbohydrate, amino acid and lipid metabolism and secondary metabolite biosynthesis. A small number of sequences involved in the transduction signal pathway were detected.

In addition to this general annotation, a further annotation restricted to published sequence data of *C. sativus* was carried out using NCBI entries from different databases (ESTs, nucleotides, and proteins) and the transcriptome published by Jain et al. (2016) derived from an Indian saffron accession. A good percentage of published sequences showed similarity with the contigs generated in the present study (Table 6). The annotation against saffron sequences was carried out not only for the reference hypomethylome but also for the five independent assemblies of the different accessions. For each independent assembly the number of contigs presenting similarity with the reference databases is reported (Table 5). The different annotations are reported in Supplementary Table 1.

**TABLE 6:** Pairwise comparison among accessions in search for differentially methylated regions.

Comparison	Regions in first^a^	Regions in second^b^
BCU001610 – BCU001637	8,470	5,743
BCU001610 – BCU001668	3,436	7,300
BCU001610 – BCU001754	10,503	5,885
BCU001610 – BCU002519	3,658	6,209
BCU001637 – BCU001668	3,085	9,682
BCU001637 – BCU001754	5,790	3,920
BCU001637 – BCU002519	3,814	9,085
BCU001668 – BCU001754	11,451	3,059
BCU001668 – BCU002519	4,916	3,626
BCU001754 – BCU002519	4,336	11,396

*A high number of differentially methylated regions was highlighted for each comparison. Only the sequences also present in the reference hypomethylome were considered for the comparison. ^a^Sequences present in the first accession and absent in the second one. ^b^Sequences present in the second accession and absent in the first one.*

In the active sheets Csativus EST, Csativus nucleotide, Csativus protein and Csativus transcriptome, the global numbers (with statistical parameters and description) of positive hits of the assembled contigs for the reference hypomethylome with the saffron sequences of the different databases are reported. For each contig, the identification of the published sequence, the identification of the contig, some statistical parameters such as the *E*-value and the description of the corresponding EST, nucleotide and protein are presented. In the comparison with the published *C. sativus* transcriptome, out of 23,256 transcriptome reads similar to hypomethylated regions, 1,418 sequences (6.1%) corresponded to transcription factors of different families like ARF, AUXIN/IAA, MADS, MYB, and WRKY, while 13,380 sequences (57.6%) corresponded to genes with a known description. When considering only genes with a known description, the percentage of transcription factors increased to 9.6%.

### Differentially Methylated Regions and Alternative Phenotypes

To check for a possible association among differentially methylated regions and the alternative phenotypes of the selected accessions, a pairwise comparison among samples was carried out. All possible pairwise comparisons were performed and a high number of differentially methylated regions was detected among accessions (Table 6), supporting the presence of high epigenetic variability among samples. The differentially methylated regions were analyzed against the available databases, focusing on those sequences coding for proteins presenting good identities with known saffron proteins. The highest number of differentially methylated regions was found in the comparisons involving the Indian accession BCU001754, as evident in BCU001754 versus the Spanish accessions BCU001610, BCU001668, and BCU002519. For each pairwise comparison, more information concerning the identity and the sequence of the differential contigs is available in Supplementary Table 4.

Similarly, contigs of the reference hypomethylome were used to search for sequences occurring uniquely in single accessions. Only sequences longer than 50 bp were considered. As evident from Table 7 and Supplementary Table 5, a discrete number of unique sequences was detected, ranging from 175 in BCU001610 to 289 in BCU001668.

**TABLE 7:** Contigs of the reference hypomethylome derived exclusively from single accessions.

Accessions	Unique regions = 50 bp
BCU001610	175
BCU001637	217
BCU001668	289
BCU001754	261
BCU002519	230

In order to determine the presence of sequences that could be associated with the alternative phenotypes, we focused on the following pairwise comparisons: (i) BCU001610 versus BCU002519, because of their different saffron yields, (ii) BCU001637 versus BCU001668, because of the different flowering time, and (iii) the unique sequences of BCU001754, because of its different pigmentation, involving darker veins in the tepals with respect to the classical tepal pigmentation.

**FIGURE 1:**
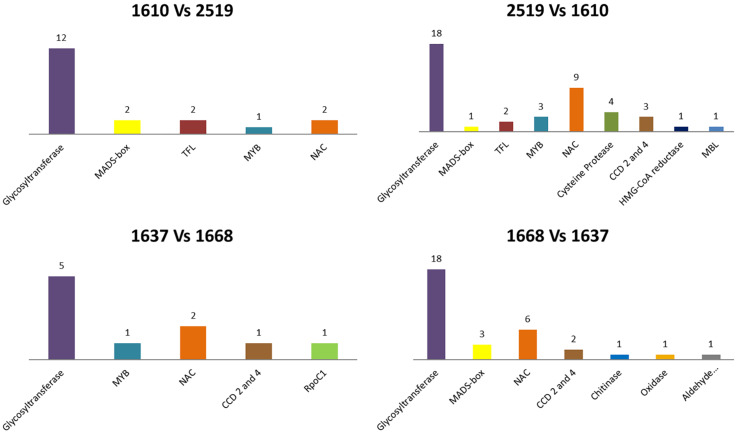
Correspondence among differentially methylated regions in the two pairwise comparisons and known saffron protein sequences available in NCBI database. (1a) sequences present only in the accession BCU001610; (1b) sequences present only in the accession BCU002519; (1c) sequences present only in the accession BCU001637; (1d) sequences present only in the accession BCU001668. The histograms represent the different kinds of proteins while their height is proportional to the number of differentially methylated regions presenting identity with that specific class of proteins.

Comparison of BCU001610 versus BCU002519 revealed that 3,658 and 6,209 sequences were differentially present in the two accessions, respectively. For BCU001610, 1,022 of the differentially methylated regions encoded for proteins and 19 of these presented a high identity to known saffron proteins. For BCU002519, 1,851 of the differentially methylated regions encoded for proteins, of which 42 presented a high identity to known saffron proteins (Figure 1). The highest number of sequences encoded for proteins showing a high identity to known saffron glycosyltransferases (12 and 18 for the two accessions, respectively). Interestingly, a certain number of proteins showed identity with known saffron transcription factors belonging to the superfamilies MADS-box, TFL (FLOWERING LOCUS T/TERMINAL), MYB and NAC (NAM, ATAF1,2 and CUC2). Among the proteins encoding for enzymes, two CCD (carotenoid cleavage dioxygenase) proteins (CCD2 and CCD4) were detected.

In the comparison between BCU001637 and BCU001668, 3,085 and 9,682 sequences were differentially present in the two accessions, respectively. For BCU001637, 10 out of 886 of the differentially methylated regions encoding for proteins corresponded to known saffron proteins. For BCU001668, 1,701 of the differentially methylated regions encoded for proteins, of which 32 presented high similarity to known saffron proteins (Figure 1). As for the previous comparison, the highest number of sequences encoded for proteins matching known saffron glycosyltransferases (5 and 18 for the two accessions, respectively) but also in this case a certain number of proteins showed identity with known saffron transcription factors belonging to the superfamilies MADS-box, TFL, MYB and NAC. Two CCD (carotenoid cleavage dioxygenase) proteins (CCD2 and CCD4) were also detected.

Finally, considering the unique sequences present in BCU001754, out of 261 unique contigs present in the reference hypomethylome and not covered by any other accession, 68 were found to correspond at least partially to genes encoding for plant proteins. Among these, six contigs presented high identities with known saffron proteins already available in online databases. Among these proteins, many transcription factors belonging to different families were identified, together with two sequences related to MYB transcription factors (a saffron MYB protein and an *Elaeis guineensis* myb-related protein MYBAS2).

### Identification of Sequence Differences Between the Accessions

**FIGURE 2:**
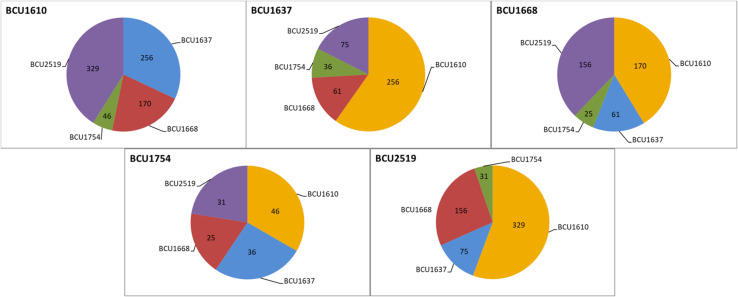
Sequence polymorphisms (SNPs and indels) among the different accessions considered in the study; the origin of the accessions is reported in Table 1.

After we had demonstrated very high epigenetic variability among the accessions, we focused on finding genetic differences (SNPs and indels) among them. Each position in the assembled contigs was evaluated for the occurrence of differences between datasets. The analysis with a minimum coverage of 10 reads in both datasets revealed on average 103 differing positions ranging from 25 (differences between BCU001668 and 1754) to 329 (differences between BCU001610 and 2519) (Figure 2). The polymorphic sites were considered as true only in the presence of a minimal difference score of 180 (Supplementary Table 6). The Indian accession presented a number of mutations with respect to the other accessions, ranging from 25 (with BCU001668) to 46 (with BCU001610), despite its completely different geographic origin. BCU001610 was characterized globally by 801 mutations, and with respect to the other accessions ranging from 46 (with BCU001754) to 329 (with BCU002519). Note that BCU001610 presented the highest number of mutations against the accession having the alternative phenotype, BCU002519.

### Functional Annotation and Polymorphism Distribution Between Gene Ontologies

We used the GO enrichment analysis to map and visualize sequence polymorphisms located within GO terms and to compare their distributions among different accessions. Using the threshold of 180 for the minimal difference score, the number of variations that occurred in genomic regions linked to GO terms ranged from 55 to 140 within 15 to 42 mapped GOs, respectively (Table 8) (more detailed results are reported in Supplementary Table 7). Considering the low number of polymorphisms detected, a good number of polymorphic GOs were evidenced. As in the previous analysis of SNP/indel frequencies, this observation confirmed that the Spanish samples had the highest number of mutations with respect to the other accessions, especially when compared to the Indian sample. Interestingly, despite having the lowest number of pathways mapped, the Indian accession had the highest number of polymorphisms within each GO. Three GO terms (GO:0008152 – metabolic process, GO:0008375 – acetylglucosaminyltransferase activity and GO:0016020 – membrane) were polymorphic within all five accessions (Supplementary Table 7). When the analysis was repeated without implementing the threshold of 180 but including all the detected polymorphisms, as expected the total number of polymorphic GOs and the corresponding numbers of polymorphisms within GO terms increased considerably (Table 8).

**TABLE 8:** Number of GOs mapped to previously identified mutations (SNPs and indels) and total number of polymorphisms associated with GOs.

Accessions	Number of polymorphic GOs^*a*^	Number of polymorphisms within GO terms^*a*^	Number of polymorphic GOs^*b*^	Number of polymorphisms within GO terms^*b*^
BCU001610	31	75	1,918	11,212
BCU001637	28	100	1,870	11,693
BCU001668	35	99	2,048	12,309
BCU001754	15	55	1,846	12,623
BCU002519	42	140	2,094	13,153

*^a^Minimal difference score of 180; ^b^all polymorphisms without implementing a threshold for the minimal difference score.*

**FIGURE 3:**
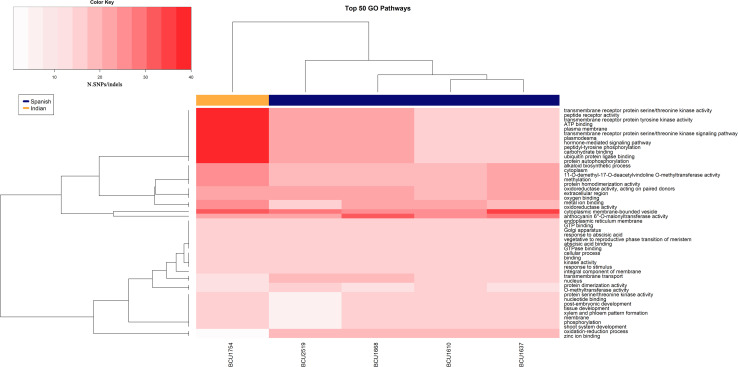
Heatmap showing the top 50 GOs with the highest value of polymorphisms.

Based on the latter data, a heatmap of the 50 highest polymorphic GOs shared between accessions (Figure 3) was obtained, highlighting the presence of two distinct clusters corresponding clearly to Spanish and Indian samples. The 12 GOs mainly driving the clusterization were involved in cell surface and cell signaling activity, with particular heterogeneity in the Indian accession.

### Population Structure and Genetic Relationship

To assess the genetic relationship among the five different accessions, we examined genome-wide single site mutation (SNPs and indels) data. We performed a PCA to examine the population structure at the level of single accession (Supplementary Figure 2). Four principal components could be extracted explaining 32.83, 27.07, 25.21, and 14.89% of the total variance, respectively. The three accessions BCU001637, BCU001668, and BCU001754, which, respectively corresponded to early and late flowering time and different pigmentation phenotype, were separated by PC1 from the other two samples linked to high/low saffron yield (BCU001610 and BCU002519). Furthermore, according to the coordinates of PC4, which explained 14.89% of the total variance, the higher number of polymorphisms in the Spanish accessions separated this group well from the Indian sample. Population structure was also analyzed in the same dataset using Admixture software for clustering solutions at *K* = 2–5 (Supplementary Figure 3). The lowest cross-validation error value, CV = 0.02558, was scored at *K* = 5. At *K* = 3, all the accessions but the two related to high/low saffron yield clustered together, confirming the same differentiation observed on the first principal component (PC1), but as K increased to 4 and 5, all studied samples revealed different combinations of ancestry components, showing an extremely heterogeneous genetic structure with barely distinguishable ancestral populations.

## Discussion

Saffron crocus is characterized by high phenotypic plasticity and variability despite a surprisingly low level of reported genetic variability given that saffron domestication started around 4000 years ago. To increase the existing knowledge on both epigenetic and genetic variability present among saffron accessions with different phenotypes, five accessions were selected for this study based on their clearly different phenotypes and low level of genetic variability detected by AFLP analysis (Busconi et al., 2015). Since the production of the spice (stigmata) is the most important character for saffron crocus, the selection of the accessions was carried out by considering alternative phenotypes related to flowering. In particular, BCU001610 and BCU002519 were included because of their similar geographic origin and flowering period but extremely different saffron spice yield, while BCU001637 and BCU001668 were selected because of their similar geographic origin and extremely different flowering period. Concerning absolute saffron production, BCU001668 yields the lowest amount of saffron among the five accessions but this trait is associated with highly delayed flowering and for that reason it could not be used as a reference for low yield. Finally, the Indian accession BCU001754, despite being the most productive accession, was selected because of its clearly different pigmentation of the tepals. Despite having a flowering period similar to that of BCU001610 and BCU002519, the Indian accession was not considered as a reference for high yield because of its different geographic origin.

The analyses were carried out by applying the MRE-seq approach, which has previously proved to be effective for a polyploid species like saffron (Wischnitzki et al., 2015), despite (1) the large genome (10.3 Gb estimated by Brandizzi and Caiola, 1998), (2) the lack of a reference genome sequence, and (3) the allotriploid origin, which introduces the complexity of heterozygosity and different allelic variants to the analysis. In the absence of a reference genome, and following the suggestion of Wischnitzki et al. (2015), the rice genome was considered as representative of monocots for sequence annotation.

*Hpa*II, the restriction enzyme used to generate the DNA fragments to be sequenced, cuts at its target site (CCGG) in the absence of methylation but is unable to cut the DNA in the presence of C methylation on both strands, while the presence of C methylation on just one strand leads to a negligible or slow nicking of the non-methylated strand (Fulneček and Kovařík, 2014). Consequently, the enzyme is very suitable for enriching hypomethylated regions of plant genomes (plant hypomethylome) that are frequently associated with transcription activity. On the contrary, cytosine methylation is found predominantly in repetitive elements (Rabinowicz et al., 2005). It is widely reported in the literature that any type of methylation in the regulatory regions, within the gene space and over the transcription start site, is frequently correlated with gene silencing (Wang et al., 2014; Niederhuth et al., 2016; Springer and Schmitz, 2017).

The herein generated sequences were of HQ, with more than 92% of sequences of each accession passing all filtration steps (Table 2). The *de novo* assembly resulted in more than 32,000 contigs, incorporating 75.8% of the sequence reads. By combining the data gathered from the five accessions, it was possible to obtain an integrated hypomethylome accounting for 43,897 contigs, including 86.2% of the sequence reads (Table 4). The obtained reference hypomethylome was then used for the annotation step and the subsequent analyses. As already described in similar studies, the coverage was low (close to the 0.2 *X*-value reported in Wischnitzki et al., 2015), thus we did not expect to have reconstructed the complete hypomethylome; however, we still found a depletion in transposable elements (less than 1% of sequences derived from repetitive elements) and an enrichment for gene-associated regions close to the percentage reported in the literature (Wischnitzki et al., 2015). Globally, 11,569 contigs, corresponding to 26.35% of the total, could be annotated as being at least partially protein coding by comparing our sequences with online resources such as the NCBI NR protein and NT nucleotide databases, the *O. sativa* genome, GO, KEGG – Kyoto Encyclopedia of Genes and Genomes and the available saffron sequences (Table 4). Interestingly, 22% of saffron sequences from a recent transcriptome sequencing project (Jain et al., 2016) showed positive hits with our sequences, further supporting the successful enrichment for coding sequences (Table 9). Genes encoding for transcription factors and enzymatic proteins involved in different metabolic pathways of primary and secondary cellular metabolism were identified. The most frequent metabolic pathways were those associated with the metabolism of amino acids, nucleotides, carbohydrates, fatty acids, secondary metabolites, and with photosynthesis.

**TABLE 9:** *C. sativus* sequences considered for the annotation.

Reference database	Number of sequences	Positive hits	Similar to hypomethylated regions	% of the total
NCBI EST	6,784	2,537	1,335	20%
NCBI nucleotide	271	163	95	35%
NCBI protein	177	1,972	93	52%
Saffron transcriptome	105,269	54,533	23,256	22%

*For each sequence typology we reported: (1) the number of sequences present in the considered database; (2) the number of contigs with positive hits with the sequences of the databases; (3) the number of published sequences similar to hypomethylated regions after removing multiple hits of the same sequence with different contigs; and (4) percentage of sequences similar to hypomethylated regions with respect to the total number of sequences.*

Subsequently, the presence of differentially methylated regions was checked in order to identify possible associations with the alternative phenotypes of interest. Despite using leaves, we identified regions that could be good candidates to explain the alternative phenotypes. Although there is a possibility that the epigenetic profile of the leaves may differ from those of the flowers, in preliminary studies carried out in our laboratory they produced very similar profiles, with only small differences.

Here two strategies were used to identify differentially methylated regions: (1) by making all possible pairwise comparisons between accessions, and (2) identifying sequences unique to single accessions. In the first case, the regions identified in the reference assembly using one accession at a time were checked to test whether they were also covered by a second accession. The regions not covered were extracted and considered as differentially methylated. In the second case, contigs of the reference hypomethylome deriving exclusively from single accessions were identified. In both cases, only regions with a minimal length of 50 bp were considered when we searched for sequences encoding proteins putatively interesting for the phenotypes under investigation. Clearly, by comparing just two accessions at a time, the number of sequences deriving from differentially methylated regions was higher than when all five accessions were compared at the same time.

As shown in Table 6, a large number of sequences deriving from differentially methylated regions were present in all the pairwise comparisons, supporting previous evidence (Busconi et al., 2015) that epigenetic variability among accessions is very high, regardless of the origin of the accession. As expected, almost all comparisons including BCU001754 presented a large number of differentially methylated regions and this could be the consequence of the different geographic origins, BCU001754 being the only Indian accession considered in this study. As reported in the materials and methods sections, the five accessions were co-cultivated closely in the same field, facing similar environmental conditions for at least 8 consecutive years and, despite this, the five accessions still presented high epigenetic variability. This indicates the high stability of the saffron epigenome over time. Prolonged cultivation in the same environment is not enough to uniform the different accessions to a similar epigenome.

Among all possible comparisons, we were mainly interested in comparing the high and low saffron yielding (BCU001610 and BCU002519) and the early and late flowering (BCU001637 and BCU001668) phenotypes. For both comparisons, a large number of differentially methylated regions encoding for proteins were detected; among these, from 10 to 42 protein-coding sequences presented high identity with known saffron proteins, including several transcription factors belonging to the MADS-box and TFL gene families. MADS-box genes encode for transcription factors that play a significant role in plant development, especially in determining floral organ identities (Zhang et al., 2017). In our study, sequences encoding for APETALA (AP1 and AP3) and AGAMOUS transcription factors were found. AP1 is involved in the establishment and maintenance of floral identity in newly formed floral primordia. AP1 and AP3 together with another MADS-box gene, PISTILLATA, regulate genes required for petal and stamen morphogenesis. AGAMOUS is responsible for the formation of stamens and carpels in the third and fourth whorls of the wild type flower. The FT/TFL gene family (FLOWERING LOCUS T, FT, and TERMINAL FLOWER 1, TFL1) is involved in the transition to and repression of reproductive development and flowering, whereby FT is able to promote the transition, whereas TFL1 represses it. In addition to flowering regulation, recent evidence in different species supports the role of these genes in other developmental processes (Wickland and Hanzawa, 2015). Given that the alternative phenotypes of the accessions under comparison were associated with flowering, it is interesting to note that in the differentially methylated regions we were able to detect the presence of sequences encoding for proteins that are extremely important for this process. Clearly, we cannot state that the transcription factors we detected are the actual cause of the observed alternative phenotypes; however, the differentially methylated regions we identified are good candidates for explaining the differences among the considered accessions.

Among the differentially methylated regions, sequences encoding for CCD (carotenoid cleavage dioxygenase) proteins (CCD2 and CCD4) were also detected. This is interesting given that these proteins are involved in the preliminary steps of the metabolic pathway for the biosynthesis of crocin, picrocrocin and safranal, starting from zeaxhantin. Crocin, picrocrocin and safranal are the three most important metabolites conferring the typical color, flavor, and aroma to the saffron spice.

Among the sequences unique to accession BCU001754, which is characterized by a different tepal pigmentation, transcription factors of the MYB family were detected. Among the flavonoids, anthocyanins are pigments resulting in pink, red, orange, scarlet, purple, blue, and blue-black flower colors. The expression of biosynthetic genes involved in the flavonoid pathway is regulated by the coordinated activity of a complex of proteins involving MYB transcription factors. Several studies have reported the involvement of MYB transcription factors in different tissue pigmentation patterns, as in *Medicago truncatula* mutants (Carletti et al., 2013). Walker et al. (2007) determined that the difference between red and white grapevine cultivars was due to mutations in two genes located in close proximity on the same chromosome and belonging to the MYB gene family. Interestingly, in white cultivars, one of the two genes, VvMYBA1, is inactivated due to the insertion of a retrotransposon in the promoter, which led to epigenetic changes resulting in the inactivation of the gene. Given the involvement of MYB transcription factors in pigment production and petal morphogenesis (Baumann et al., 2007) we speculate that the identified region is a good candidate for causing the alternative phenotype of BCU001754.

When we used the generated sequence data to highlight the presence of mutations at the DNA level, a discrete number of single-base genetic mutations, SNPs and INDELs was observed among accessions, contrary to preliminary studies in which low (Busconi et al., 2015) or even no (Rubio Moraga et al., 2009) genetic variation was reported. This difference between studies may be due to the different throughput of the molecular techniques adopted, with massive sequencing providing more information than the application of classical molecular markers such as RAPDs or AFLPs. The presence of these genetic mutations prompted the search for mutations in genes encoding for proteins and the identification of alternative phenotypes with genetic and not only epigenetic bases. A search for polymorphisms located within GO terms was carried out revealing from 15 to 42 polymorphic GO terms, three of which were polymorphic in all five accessions. Finding evidence of the presence of polymorphic GOs, despite the low number of variants detected with the threshold of 180, is a consequence of focusing the analysis on gene-associated regions. Subsequently, all the identified SNPs, including those with a minimal score lower than 180, were analyzed for sequence polymorphisms located within GO terms, and their distributions were compared among different accessions. Once polymorphic GOs had been identified, the next step was to analyze the distribution of the most polymorphic GO among the five accessions. The heat map in Figure 3 shows two clusters separating the accessions based on their geographic origin. In particular, a group of 12 GOs mainly involved in cell surface and cell signaling activity presented a higher polymorphism in the Indian accession with respect to the Spanish ones. The difference is evident, and this pattern could be considered as a kind of signature of selection in the Indian accession with respect to the Spanish ones. The possibility that spontaneous mutations that increase the fitness of the populations in local environmental conditions are selected over time by farmers has been hypothesized by scientists working on saffron. Our findings could be the first evidence supporting this hypothesis, but need to be confirmed by analyzing further Spanish and Indian accessions based on the genetic markers located in these polymorphic GOs. Using all polymorphisms, regardless of the score obtained, on the one hand increases the risk of including sequence artifacts in the analysis, but on the other hand can lead to more information about true polymorphisms that might be excluded when a high score threshold is applied. The results, as shown in the heat map, seem to support the use of all polymorphisms.

Finally, the analysis of population structure and genetic relationships among accessions based on the genetic polymorphisms showed that BCU001754 can be separated from the other accessions (Supplementary Figure 2, PC1 Vs PC4 and PC2 Vs PC4). Within Spanish accessions, the differentiation of the samples based on the province of origin was not as clear. Similarly, the differentiation of the other variables (saffron yield and flowering time) was not as evident as for the geographic origin, but some differences were apparent: the accession with high saffron yield could be separated from the accession with low yield and the same was true for flowering time. Similar information was obtained from the structure analysis. Taken together these results strongly support the existence of variability at the genetic level, likely deriving from spontaneous mutations that occurred during the continuous vegetative propagation of this sterile species. Further, we obtained evidence that (1) variation (SNPs/indels) can be used to differentiate the accessions based on their origin, (2) the genetic variants can be close or within protein coding sequences, supporting alternative phenotypes as the result of genetic and not only epigenetic variability, and (3) the genetic variants can be selected by humans in different growing areas, resulting in local populations with alternative phenotypes.

## Conclusion

This study represents the first high throughput analysis carried out to extend existing knowledge on saffron epigenetics. Despite the low coverage obtained from MRE-seq due to the huge genome size of the species, interesting information was collected. Among five phenotypically different accessions, differentially methylated regions were detected corresponding to genes encoding for proteins, such as transcription factors, that could be involved in shaping the alternative phenotypes. By comparing the generated sequences, a high number of genetic variants, SNPs and INDELs were identified, definitively ending the debate concerning the presence/absence of genetic polymorphisms within the species. Many genetic variants were also detected in GO terms, suggesting the existence of a genetic basis for alternative phenotypes. A group of 12 GOs presented higher polymorphism in the Indian compared to the Spanish accessions and this points toward evidence of selection carried out by farmers and/or environmental conditions in the different growing areas.

## Data Availability Statement

The raw reads of Methylation-sensitive restriction enzyme sequencing (MRE-seq) have been deposited in NCBI Sequence Read Archive with accession numbers GSE166350.

## Author Contributions

MB, SF, MM, and JF contributed to design of the experiment. MB, GS, and ES carried out the experiment. EW, MD, LS, and LC performed bioinformatic analysis. MM and JF selected and provided the sample of saffron for the analysis. MB, LC, LS, and JF interpreted the results. MB, GS, and JF wrote the article. MB, LC, ES, MM, and JF revised the article. All authors contributed to the article and approved the submitted version.

## Conflict of Interest

The authors declare that the research was conducted in the absence of any commercial or financial relationships that could be construed as a potential conflict of interest.

## Footnotes

1 www.genome.jp/kegg/pathway.html

2 http://broadinstitute.github.io/picard

## Supplementary Material

Supplementary Figure 1 Length distribution of *de novo* assembled contigs for the single accessions and for the combined reference hypomethylome.

Supplementary Figure 2 Principal component analysis results. The four components explaining 100% of the variance were plotted. The five accessions are represented by different colors. Plots corresponding to PC1-PC4 and PC2-PC4 make it possible to distinguish the accessions based on their geographic origin.

Supplementary Figure 3 Population structure analysis of the five samples for K values from *K* = 2 to 5. Each accession is represented by a vertical column, which is partitioned into colored segments that represent the proportion of the inferred K clusters.

Supplementary Table 1 Phenotypic evaluations of the five accessions focusing on saffron production and flowering time. BCU001668 clearly started flowering after the other accessions. There was no significant difference among the other accessions in relation to this trait. BCU001610 was very productive in the flowering period, however, the most productive accession was BCU001754. BCU002519 produced low saffron yields in the flowering period, but the lowest yield was observed in accession BCU001668.

Supplementary Table 2 Annotation results for the sequences of the reference hypomethylome against the different online databases. The active sheets report, respectively: NR and NT, the annotation against the non-redundant protein sequences (NR) and nucleotide (NT) databases; *O. sativa* genome, the annotation against the rice genome (the best annotated monocot genome); GO and KEGG, information, for protein coding sequences, about GO terms and KEGG pathway. Finally, the annotation against available sequences of *C. sativus* is reported. Alignment was carried out by considering both the forward and reverse sequences. For the EST, nucleotide and protein databases we report the different statistics of the alignment and the description, if available, of the gene products and of the protein function. For the annotation against the available transcriptome, as well as the gene description, we report: (i) whether the gene encodes for a transcription factor, (ii) the EuKaryotic Orthologous Groups (KOG class), and (iii) the GO annotation.

Supplementary Table 3 The results of the analysis against the KEGG database are reported. The different sequences are ordered based on the KEGG pathways: lipid metabolism, carbohydrate metabolism, nucleic acid metabolism and so on. For each pathway the corresponding map is reported. The most represented pathways were those of carbohydrate, amino acid and lipid metabolism and secondary metabolite biosynthesis.

Supplementary Table 4 The differentially methylated sequences identified in all the possible pairwise comparisons are reported.

Supplementary Table 5 The unique sequences identified in single accessions are reported.

Supplementary Table 6 For each pairwise comparison, the different polymorphisms, SNPs and indels characterized by a minimal difference score higher than 180, are reported. The detected alternative in the accessions under comparison is indicated.

Supplementary Table 7 For each accession, the GO description and the GO identity are reported. Only those sequences presenting a minimal difference score higher than 180 were considered.
