# Supplementary figures and images for: Epigenetic Variability Among Saffron Crocus (Crocus sativus L.) Accessions Characterized by Different Phenotypes
Source: Front Plant Sci. 2021 Mar 4;12:642631. doi: 10.3389/fpls.2021.642631 (PMC7970008; doi:10.3389/fpls.2021.642631)

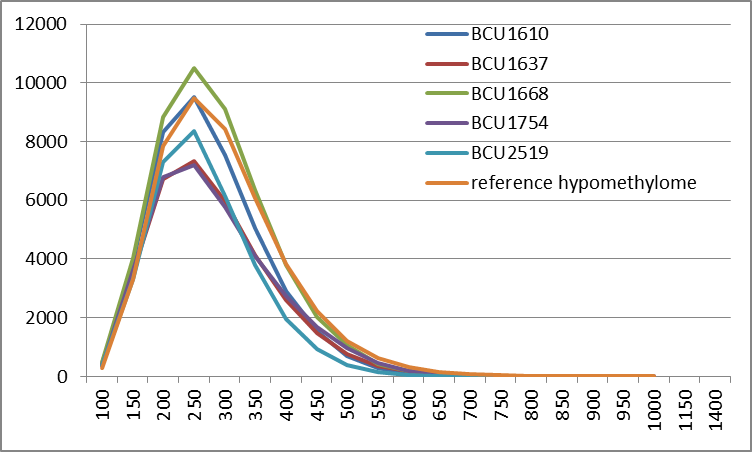

Supplement: Supplementary Figure 1 — Length distribution of de novo assembled contigs for the single accessions and for the combined reference hypomethylome. [file Image_1.TIF]

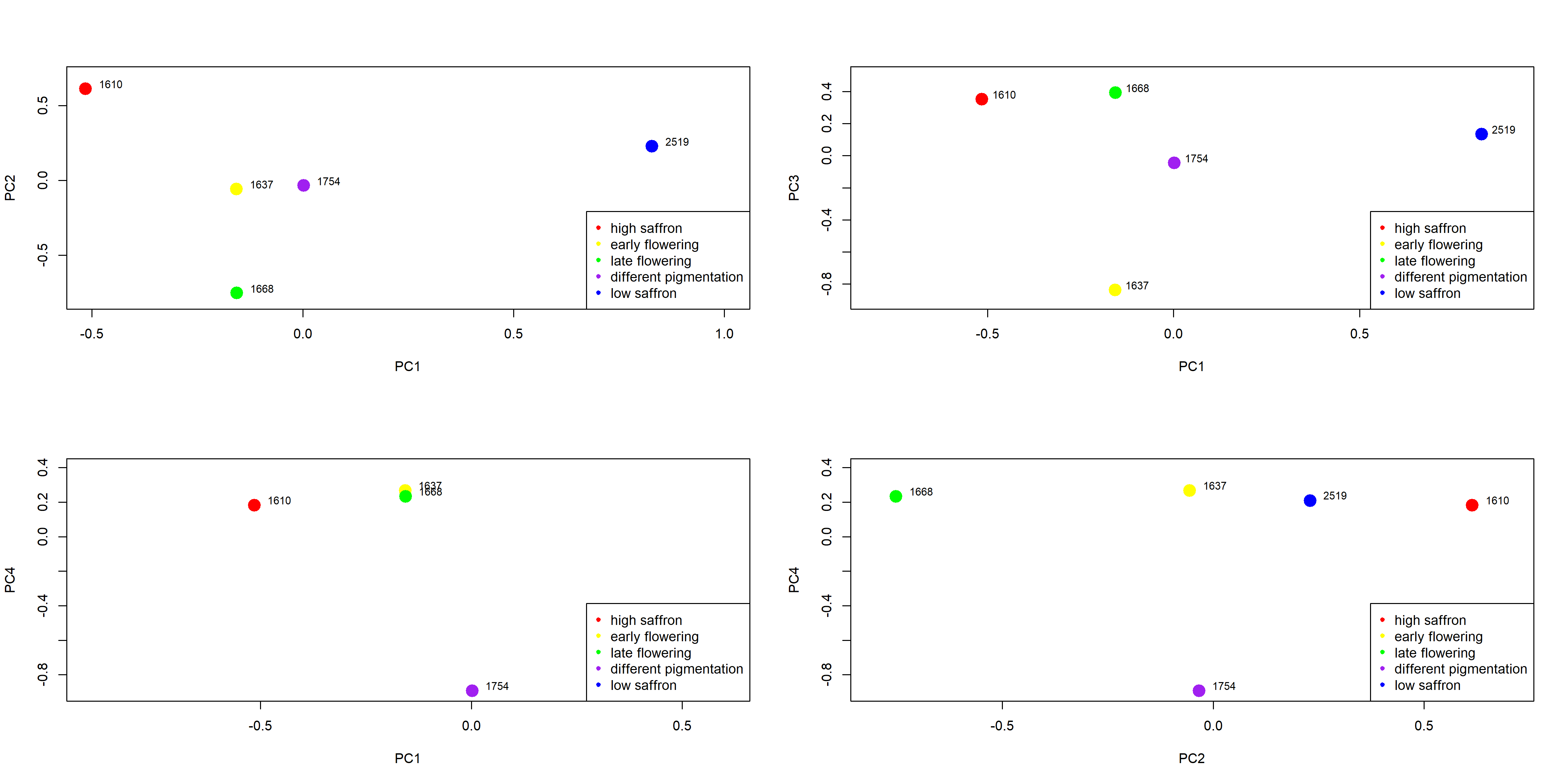

Supplement: Supplementary Figure 2 — Principal component analysis results. The four components explaining 100% of the variance were plotted. The five accessions are represented by different colors. Plots corresponding to PC1-PC4 and PC2-PC4 make it possible to distinguish the accessions based on their geographic origin. [file Image_2.TIFF]

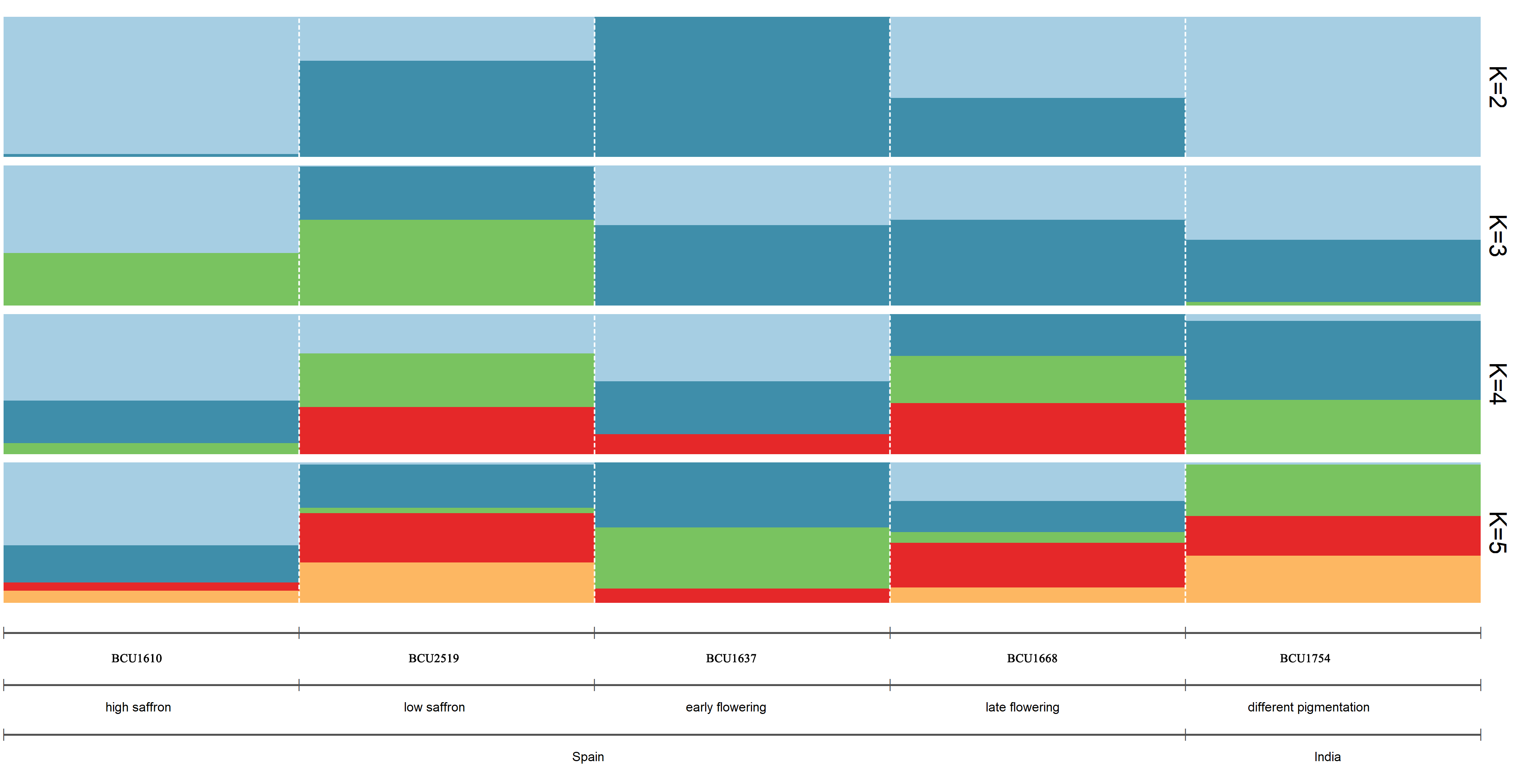

Supplement: Supplementary Figure 3 — Population structure analysis of the five samples for K values from K = 2 to 5. Each accession is represented by a vertical column, which is partitioned into colored segments that represent the proportion of the inferred K clusters. [file Image_3.TIFF]
