# Supplementary material for: Epigenetic Variability Among Saffron Crocus (Crocus sativus L.) Accessions Characterized by Different Phenotypes
Source: Front Plant Sci. 2021 Mar 4;12:642631. doi: 10.3389/fpls.2021.642631 (PMC7970008; doi:10.3389/fpls.2021.642631)
Supplement: Supplementary Table 1 — Phenotypic evaluations of the five accessions focusing on saffron production and flowering time. BCU001668 clearly started flowering after the other accessions. There was no significant difference among the other accessions in relation to this trait. BCU001610 was very productive in the flowering period, however, the most productive accession was BCU001754. BCU002519 produced low saffron yields in the flowering period, but the lowest yield was observed in accession BCU001668. [file Table_1.DOCX]

**Supplementary table 1.** Phenotypic evaluations of the five accessions focusing on saffron production and flowering time. BCU001668 clearly started flowering after the other accessions. There was no significant difference among the other accessions in relation to this trait. BCU001610 was very productive in the flowering period, however the most productive accession was BCU001754. BCU002519 produced low saffron yields in the flowering period, but the lowest yield was observed in accession BCU001668.

| **Accession** | **Dry weight saffron per plant (mg)^a^** | **SD^b^** | **Days to flowering** | **SD** |
| --- | --- | --- | --- | --- |
| BCU001610 | 27.33 | 10.09 | 48.67 | 2.31 |
| BCU002519 | 10.78 | 8.43 | 48.33 | 2.08 |
| BCU001637 | 23.92 | 7.28 | 46.67 | 1.15 |
| BCU001668 | 2.93 | 0.62 | 53.33 | 1.15 |
| BCU001754 | 35.33 | 8.05 | 49.00 | 2.65 |

a: Average value of three replicates for accession; b: Standard deviation
